# Influence of psychosocial risk factors on the trajectory of mental health problems from childhood to adolescence: a longitudinal study

**DOI:** 10.1186/1471-244X-13-31

**Published:** 2013-01-17

**Authors:** Daniel Fatori, Isabel A Bordin, Bartira M Curto, Cristiane S de Paula

**Affiliations:** 1 Institute of Psychiatry, University of Sao Paulo Medical School, Sao Paulo, Brazil; 2 Social Psychiatry Division, Federal University of São Paulo, Sao Paulo, Brazil; 3 Developmental Disorder Post Graduation Program, Mackenzie Presbyterian University, Sao Paulo, Brazil

**Keywords:** Child, Adolescent, Violence, Epidemiology, Longitudinal studies, Psychopathology, Risk factors, Mental health, Developing countries

## Abstract

**Background:**

Longitudinal epidemiological studies involving child/adolescent mental health problems are scarce in developing countries, particularly in regions characterized by adverse living conditions. We examined the influence of psychosocial factors on the trajectory of child/adolescent mental health problems (CAMHP) over time.

**Methods:**

A population-based sample of 6- to 13-year-olds with CAMHP was followed-up from 2002–2003 (Time 1/T1) to 2007–2008 (Time 2/T2), with 86 out of 124 eligible children/adolescents at T1 being reassessed at T2 (sample loss: 30.6%). Outcome: CAMHP at T2 according to the Child Behavior Checklist/CBCL’s total problem scale. Psychosocial factors: T1 variables (child/adolescent’s age, family socioeconomic status); trajectory of variables from T1 to T2 (child/adolescent exposure to severe physical punishment, mother exposure to severe physical marital violence, maternal anxiety/depression); and T2 variables (maternal education, child/adolescent’s social support and pro-social activities).

**Results:**

Multivariate analysis identified two risk factors for child/adolescent MHP at T2: aggravation of child/adolescent physical punishment and aggravation of maternal anxiety/depression.

**Conclusions:**

The current study shows the importance of considering child/adolescent physical punishment and maternal anxiety/depression in intervention models and mental health care policies.

## Background

Child and adolescent mental health problems (CAMHP) are prevalent worldwide: approximately 12% of youth have a mental disorder [1]. Even though reports of CAMHP prevalence rates greatly vary among developing and developed countries across the globe, recent systematic reviews clarify that differences occur mainly as a result of methodological characteristics of epidemiological studies [2,3]. Furthermore, mental health problems are a leading cause of disability in children and adolescents worldwide [4], causing enormous economic costs to society as a whole [5,6].

Currently, in order to understand the onset, course and factors associated with CAMHP there is a need to adopt a developmental perspective based on longitudinal studies [7,8]. Developmental data regarding risk and protective factors associated with CAMHP is essential to the planning of intervention models and policies; as these factors can provide a basis for treatment strategies and policy guidelines. CAMHP risk and protective factors vary across countries, especially between developing and developed countries, thus generating a need for the gathering of epidemiological data worldwide.

Findings from epidemiological studies conducted in developing countries from different continents present a diversity of risk factors associated with CAMHP. In Africa, the factors are gender, child academic ability, living with a single parent and community violence [9,10]; in Central America, the factors are gender, age, witnessing physical violence, family drug involvement, negative family interaction, school disengagement and peer deviance [11]; in Asia: gender, living in slums, child physical abuse, exposure to family violence, family involved in a major conflict, impaired reading and vocabulary, school failure, parental education, socioeconomic status (SES), academic ability, exposure to marital violence, close family member with alcohol problems and maternal anxiety/depression [12-15]. In Brazil (South America), poverty, SES, maternal anxiety/depression, child physical punishment, family trauma, exposure to marital violence and child labor were associated with CAMHP [16-21]. It is noteworthy that exposure to physical violence and poverty (low SES) are a common set of risk factors amongst developing countries, adding robustness to these findings.

The literature regarding the protective factors of CAMHP is less robust. Epidemiological data in developing countries shows association between CAMHP and protective factors such as, belief in God, parental religiosity [11], improved family life [22] and school connectedness [23]. In developed countries, the literature highlights certain CAMHP protective factors such as self-efficacy, optimism, satisfaction, self-concept, family atmosphere, parental support, peer competence [24], perceived parent and family connectedness [25], as well as social support and positive life events [26]. Few studies conducted in developing countries focused on CAMHP protective factors, making it difficult to understand differences between countries. The scarcity of such data in developing countries has been reported previously in a review of the subject [27].

The majority of findings regarding CAMHP risk and protective factors from developing countries comes from cross-sectional studies. It is difficult to understand the nature of the relationship between factors and outcomes using a cross-sectional approach. In addition, it is important to consider the trajectory of mental health problems symptoms over time and its possible interaction with outcomes. Therefore, to better comprehend this matter it is essential to conduct longitudinal studies.

In order to develop culturally appropriate interventions and policies it is essential to gather epidemiological data from developing countries, particularly in regions characterized by adverse living conditions [27,28]. While aiming to fill these gaps, the current study examines the influence of potential psychosocial risk and protective factors in terms of the aggravation of CAMHP over a period of five years in a low-income urban community. The hypothesis of the present study is that psychosocial risk factors will influence the aggravation of CAMHP over time, but protective factors will buffer its effects at least to some extent, contributing to a better prognosis.

## Methods

### Design and procedures

An epidemiological study conducted in the municipality of Embu, in the metropolitan area of the City of São Paulo, Southeastern Brazil, investigated CAMPH in a representative population-based sample of 6 to 13-year-olds (N = 345) from 2002–2003 (Time 1/T1) to 2007/2008 (Time 2/T2).

Embu is predominantly urban, characterized by neighbourhoods of small households and slums, and is located within the boundaries of the greater city of São Paulo area. Embu had an estimated population of 232,165 in 2002 and 229,327 in 2007. 38.1% of its inhabitants are under 20 years of age. Almost all children are enrolled in primary education (96.7%, years 2002 and 2007). Embu is considered a low-income community due to only 4.4% of its families having an income of more than 10 minimum wages in 2002, a low rate in comparison to the State of Sao Paulo in the same year (14.3%).

At T1 it was considered one of the most violent municipalities in the country. Homicide rates were 100.7 cases per 100.000 inhabitants in 2002, greater than the city of Sao Paulo rate of 38.9/100.000 in the same year. Five years later, the homicide rate dropped significantly (26.42/100.000), although it remained greater than that of the city of Sao Paulo (16/100.000) [29]. Human development index (HDI) of Embu is 0.772, whereas in the city of Sao Paulo the HDI is considerably higher (0.841) [30]. A previous report revealed a CAMHP prevalence of 24.6% in Embu [31].

In 2002/2003 (T1), one mother–child pair per household was randomly selected from a probabilistic sample of clusters that included all eligible households in the municipality of Embu (women aged 15–49 years with a son or daughter younger than 18 years). This population-based sample (n = 813; response rate: 82.4%) included 328 children aged 0–5 years, 349 children/adolescents aged 6–13 years, and 136 adolescents aged 14–17 years. More details regarding T1 are available elsewhere [21,31,32].

At T1, 124 participants (36%) had mental health problems according to the Child Behavior Checklist (CBCL) total problem scale (T-scores in the clinical/borderline range). At T2, 93 out of 124 children/adolescents with mental health problems at T1 were reassessed using the same instruments applied at T1. However, seven subjects among these 93 cases had to be excluded from the analysis due to missing data at T2 on study variables of interest (sample loss: 30.6%). Mothers and adolescents were individually interviewed at the local health center, and all instruments were applied by trained interviewers.

### Instruments

Child Behavior Checklist (CBCL/6-18): a standardized parent-report screening questionnaire used to identify emotional/behavioral problems in children/adolescents. Validation data for the Brazilian version of the CBCL showed adequate psychometric properties [33,34].

WorldSAFE Core Questionnaire: a standardized instrument used to investigate intrafamilial violence and risk factors. It was developed by the WorldSAFE steering committee and the Brazilian version was developed by Bordin and collaborators.

*This questionnaire includes 33 items representing various childrearing behaviors from caregivers in the past 12 month*s. Each item can be answered with a 3-point scale: not at all, 1–2 times, 3 times. Parental behaviors are based on items from the Parent–Child Conflict Tactics Scales (included with the authors’ permission) [35]. The present study’s version was translated to Portuguese and back translated to confirm the quality of the translation, as well as being field tested and applied in a pilot study [36,37].

Self-Report Questionnaire (SRQ): a screening questionnaire with 20 items developed to identify symptoms that may be indicative of mental disorders. Each item is a question with a possible answer of ‘yes’ or ‘no’. The current version detects probable cases of anxiety/depression. The SRQ is widely used in developing countries, and it was validated in Brazil, showing good validity and high reliability. Subjects scoring above 7 points are considered to be cases [38].

The Economic Classification Questionnaire was developed by the Brazilian Association of Research Companies to determine socioeconomic classes according to family purchasing power [39]. Scoring is based on the number of home appliances, the existence of private bathrooms inside or outside the residence, the educational level of the head of the household, and number of household employees working at least 5 days a week. A score between zero and 10 is indicates a low-income family and 11–34 indicates a middle/high-income family.

Protective factors questionnaire: a brief structured questionnaire about potential protective factors for CAMHP, developed by the investigators and used in a previous epidemiological study [20]. The questionnaire is comprised of 7 items developed to measure adolescent social support (family, friends/peers, school/work and community) and engagement in pro-social activity (sports, leisure and religious).

### Ethical procedures

This study was approved by the research ethics committees of the *Universidade Federal de São Paulo* (Federal University of São Paulo) and the *Universidade Presbiteriana Mackenzie* (Mackenzie Presbyterian University), and all participants have signed informed consent forms. Mothers and children/adolescents with mental health problems, at risk for suicide or victims of domestic violence were referred to specialized public services.

### Variables

(1) *Outcome*: CAMHP at T2, defined as the raw score of CBCL/6-18 total problem scale.

(2) Risk factors

(a) Trajectory of child with severe physical punishment: at T1 and T2, a continuous variable measured the number of behaviors of severe physical punishment on behalf of the parents which occurred in the past 12 months: (1) hit buttocks with an object such as a stick/broom/cane/belt; (2) hit elsewhere other than buttocks with an object; (3) choke with hands or pillow; (4) smother with hands or object around the neck; (5) kick; (6) burn/scald/brand; (7) beat (i.e. hit repeatedly with an object/fist); (8) threaten with a weapon such as knife/gun. Each item was classified as a dichotomous variable: severe physical punishment not present or severe physical punishment present at least once; then, two 0–8 scales comprised of severe physical punishment behaviors at T1 and T2 were composed. Trajectory of child severe physical punishment = T2 value - T1 value.

(b) Trajectory of marital severe physical violence against women: at T1 and T2, a continuous variable (0–4 scale) identified violent behaviors perpetrated by the mother’s resident husband/partner in the past 12 months: (1) kick; (2) hit; (3) beat; (4) threaten/assault with a weapon gun. Each item was classified in a dichotomous variable: marital severe physical violence not present or marital severe physical violence present at least once; then, two 0–7 scales were composed with marital severe physical violence behaviors at T1 and T2. Trajectory of marital severe physical violence against women = T2 value - T1 value.

(c) Trajectory of maternal anxiety/depression: measured by subtracting SRQ total score at T2 from SRQ total score at T1.

(d) Maternal education (T1): last school grade completed by the mother (continuous variable).

(e) Family socioeconomic status (T1): total score from the “Economic Classification Questionnaire” obtained at T1 (continuous variable).

(f) Child/adolescent gender.

(g) Child/adolescent age (at T1, continuous variable)

(3) *Protective factors* (T2)

(a) Social support: children/adolescents were asked if they could count on family, friends/peers, school/work and community/neighborhood. Four possible answers (very much, a little/could be better, very little, none) were dichotomized in very much (=1) versus other (=0). The sum of scores for the four social groups (0–4) defined social support.

(b) Pro-social activities: children/adolescents informed the frequency of participation in social activities: religious [once a week (=1) vs. less (=0)], sports [frequently (=1) vs. non-frequent (=0)] and other leisure activities [frequently (=1) vs. non-frequent (=0)]. The sum of scores for the three types of activities (0–3) defined participation in pro-social activities.

(4) *Control variable*: CAMHP at T1, defined as the raw score of CBCL/6-18 total problem scale.

### Statistical analysis

A multiple linear regression was performed using the backward stepwise regression method. First, all independent variables of interest were forced to enter the initial model. Then, non-significant variables were manually excluded from the model, one by one, according to the highest p value in each step, until only statistically significant variables (p < 0.05) remained in the final model. All variables were checked for multicollinearity and heteroscedasticity and none of these characteristics were present.

## Results

The mean age of children/adolescents was 9.2 ± 2.9 at T1 and 14.1 ± 2.2 at T2. At T2, 37 (39.8%) males and 56 (60.2%) females were reassessed. At T1, the mean score for family socioeconomic status was 11.6 ± 3.7, and the mean number of years for maternal education was 6.6 ± 3.2 (Table 1). At T2, 50% of mothers presented an increase in anxiety/depression symptoms and 5.8% reported an aggravation of marital severe physical violence. Also, 22% of children/adolescents presented an aggravation of severe physical punishment over time. At T2, 2.3% and 11.6% of children/adolescents reported best possible social support and pro-social activities, respectively.

**Table 1:** Means and standard deviations of demographic characteristics, CAMHP and maternal anxiety/depression at T1 and T2 in Embu, Sao Paulo, Brazil (N = 86)

	**T1**	**T2**
**Child/adolescent age**	9.2 + 2.9	14.1 ± 2.2
**CAMHP**	55.02 ± 13.41	41.56 ± 20.59
**SES**	11.6 ± 3.7	12.45 ± 3.31
**Maternal education**	6.6 ± 3.2	7.31 ± 3.58
**Maternal anxiety/depression**	7.72 ± 3.86	6.91 ± 4.34

Multivariate analysis identified two risk factors for CAMHP at T2: aggravation of the child’s physical punishment (p = 0.009) and aggravation of maternal anxiety/depression (p = 0.056) (Table 2). Even though statistical significance of aggravation of maternal anxiety/depression was marginal, it was kept in the final model to preserve goodness of fit, considering that its removal would substantially reduce the R^2^ value (from 17% to 13%). CAMHP at T1 remained in the final model as a control variable (p = 0.003).

**Table 2:** Initial and final linear regression models identifying psychosocial risk and protective factors associated with the aggravation of child and adolescent mental health problems (CAMHP) in Embu, Sao Paulo, Brazil (N = 86)

	**Initial Model**					**Final Model**				
	**B**	**SE**	***p***	**CI 95%**	**VIF**	**B**	**SE**	***p***	**CI 95%**	**VIF**
**Constant**	24.2	14.9	0.111	−5.7 – 54	–	17.9	8.4	0.036	1.2 – 34.5	–
Child/adolescent male gender	3.3	4.5	0.458	−5.6 – 12.3	1.117	–	–	–	–	–
Child/adolescent age (T1)	−1.1	1.0	0.291	−3.1 – 0.9	1.175	–	–	–	–	–
Maternal education (T1)	1.1	0.7	0.124	−0.3 – 2.5	1.243	–	–	–	–	–
Family socioeconomic status (T1)	−0.6	0.6	0.300	−1.8 – 0.5	1.154	–	–	–	–	–
Trajectory of child severe physical punishment	5.9	2.6	0.029	0.6 – 11.2	1.073	6.4	2.4	0.009	1.6 – 11	1.016
Trajectory of severe physical marital violence	0.6	4.2	0.888	−7.7 – 8.9	1.193	–	–	–	–	–
Trajectory of maternal anxiety/depression	1	0.6	0.102	−0.2 – 2.2	1.115	1.1	0.5	0.056	−0.03 – 2.2	1.001
Social support (T2)	−1.2	2.3	0.620	−5.9 – 3.5	1.162	–	–	–	–	–
Pro-social activities (T2)	−1.4	2.4	0.559	−6.2 – 3.4	1.297	–	–	–	–	–
CAMHP (T1)	0.5	0.1	0.002	0.2 – 0.9	1.209	0.4	0.1	0.003	0.1 – 0.7	1.015
R^2^	22%					17%				

## Discussion

Our explanatory model identified the influence of two risk factors in the aggravation of CAMHP from childhood to adolescence, independent of the severity of symptoms at T1: aggravation of child physical punishment and aggravation of maternal anxiety/depression. Considering the scarcity of longitudinal data in developing countries, such as Brazil, current results add important data to the literature on CAMHP psychosocial risk factors.

Cross-sectional surveys have shown that physical punishment of children is associated with CAMHP [40], but the current study demonstrates that its aggravation over time is related to the aggravation of CAMHP as time progresses. When considering the aggravation of physical punishment of a child over time as chronic violence and its association with CAMHP, our findings are consistent with previous longitudinal data [41,42]. Furthermore, it is reasonable to hypothesize that the aggravation of a child’s physical punishment over time intensifies the deleterious effect of chronic violence, consequently increasing CAMHP. In addition, this process may have inhibited the positive influence of existing social support and pro-social activities, being one of the reasons for the lack of association between protective factors at T2 and the non-aggravation of CAMHP over time, in the current study.

Maternal anxiety/depression is a well-known risk factor for CAMHP in the general population [21,32]. In the last decade, Brazilian longitudinal studies also identified this association [16,43]. Additionally, it is important to take into account the trajectory of maternal anxiety/depression symptoms, as previous studies showed that different pathways discriminate child/adolescent outcomes. For instance, higher levels of depression in adolescents were showed to be related to the maternal chronic trajectory of depression in comparison to adolescents with mothers with no depression [44]. Thus, the current study contributes new information to the field, showing a pathway where an increase in maternal anxiety/depression symptoms over time is associated with the aggravation of CAMHP.

It is worth mentioning that both the psychosocial risk factors found to be associated with CAMHP in the present study have been previously reported in studies conducted in developing and developed countries. Although in this case, given the strength of the longitudinal design of the present study, the directional relationship between factors and outcomes are elucidated.

In summary, the data provided by the current study demonstrates how important it is for clinicians to consider the trajectory of risk factors over time, especially in primary care settings in low-income communities. Also, the mother-child dyad should be prioritized in mental health care settings. When evaluating children and adolescents it is important to actively investigate physical punishment of the child and its trajectory over time. At the same time, mothers with anxiety/depression symptoms should be periodically reassessed to determine the trajectory of symptom(s) in order to detect anxiety/depression aggravation early on.

Although, a few study limitations must be recognized: (a) a small sample size that reduced power of statistical analysis; (b) sample loss of 30.7% from T1 to T2 which may have resulted in selection bias; (c) lack of T1 information on protective factors which prohibited the evaluation of the influence of respective trajectories on the study’s outcome; and (d) rates of physical violence and CAMHP may have been underreported due to parent-report measures.

## Conclusions

The current study presented an explanatory model for the aggravation of CAMHP from childhood to adolescence. The trajectory of severe physical punishment and of maternal anxiety/depression over the course of five years was associated with CAMHP aggravation. It is possible to begin development of preliminary intervention/prevention models, as well as clinician guidelines, in developing countries based on data provided by this study in tandem with other studies on risk and protective factors associated with CAMHP. Nevertheless, more longitudinal studies that could bring innovations to the field of CAMHP are still needed in developing countries to further expand current knowledge and favor more effective intervention/prevention models and guidelines that could attend mental health needs of different populations of children and adolescents worldwide.

## Competing interests

The authors declare that they have no competing interests.

## Authors’ contributions

DF drafted the manuscript, undertook statistical analysis, revision of literature and contributed directly to the final version of the manuscript. BMC coordinated field work of the follow-up study (T2) and contributed to interpretation of data. IAB designed the original study (T1) and led data acquisition. Also aided interpretation of data and revised all versions of the manuscript. CSP designed the follow-up study (T2) and led data acquisition, analysis and interpretation of the data, as well as aided the revision of the final manuscript. All authors read and approved the final manuscript.

## Pre-publication history

The pre-publication history for this paper can be accessed here:

http://www.biomedcentral.com/1471-244X/13/31/prepub
